# The important regulatory role of glucose concentration in the maturation of stem cell-derived cardiomyocytes: A review

**DOI:** 10.1097/MD.0000000000043878

**Published:** 2025-08-15

**Authors:** Liqun Chi, Junsheng Mu

**Affiliations:** a Department of Cardiac Surgery, Beijing Anzhen Hospital, Capital Medical University, Beijing Institute of Heart Lung and Blood Vessel Diseases, Beijing, China; b The Third Affilited Hospital of XinXiang Medical University, XinXiang, China.

**Keywords:** cardiomyocyte metabolism, glucose, maturation, myocardial regeneration, stem cell-derived cardiomyocyte

## Abstract

The phenotype of stem cell-derived cardiomyocytes is far from that of adult cardiomyocytes. Specifically, it is characterized by spontaneous contraction, irregular morphology, and differences in sarcomere components and metabolism. Human cardiomyocyte maturation involves a shift in metabolism from glycolysis to fatty acid oxidation. This metabolic shift alters gene expression and inhibits proliferation. These findings indicate that the glucose concentration manipulates cardiomyocyte metabolism and modulates maturation. This review summarizes the main phenotypic differences, focusing on changes in myocardial cell metabolism. We also summarize the effect of the glucose concentration on maturity of stem cell-derived cardiomyocytes, and how glucose may support a novel maturation strategy.

## 1. Introduction

Maturation of the heart is an intricate and meticulous process. It encompasses congregation, amalgamation, and interplay of diverse cell types, representing an organic integration of multiple cellular activities in a specific spatiotemporal context.^[1]^ Although scientific advances have generated stem cell-derived cardiomyocytes (CMs) from various sources,^[2]^ it remains difficult to replicate a powerful and efficient pump such as the heart. Cardiomyocytes exit the cell cycle at birth, and no evidence indicates that damaged cardiomyocytes regenerate.^[3]^ Therefore, irreversible damage to cardiomyocytes leading to heart failure is a significant public health issue. Novel therapies are important in clinical medicine.

As a novel therapy, CM transplantation has potential application prospects.^[4]^ By grafting CMs onto damaged heart tissue, we can aid myocardial repair and restore cardiac functions.^[3]^ However, disparities in the form, structure, metabolism, electrophysiology, contractility, and other aspects exist between CMs and adult cardiomyocytes.^[5]^ These differences constrain the use of CMs in disease modeling and regenerative medicine. Despite extensive scientific studies to enhance CM maturation via physical, chemical, genomic, and biological approaches, bridging these differences remains challenging.^[6–10]^ Thus, accelerating CM maturation in a rational timeframe poses a challenge.

Maturation of human cardiomyocytes involves their metabolism shifting from glycolysis to fatty acid oxidation.^[11,12]^ This metabolic shift alters gene expression and inhibits proliferation.^[13]^ These findings indicate that the glucose concentration manipulates cardiomyocyte metabolism and modulates maturation.^[5]^ This review discusses how the glucose concentration affects CM maturation to provide a novel theoretical basis and experimental methods to promote CM maturity.

## 2. CMs versus adult cardiomyocytes

**Figure 1.**
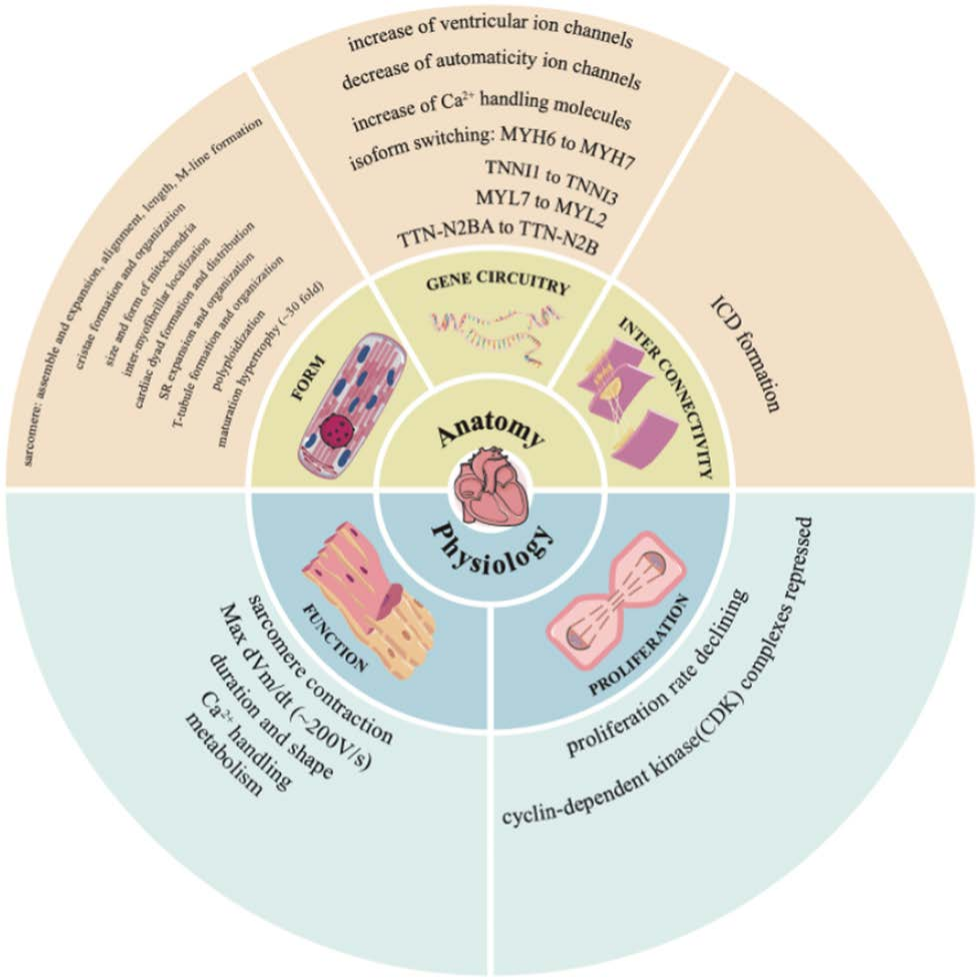
The differences between CMs and adult cardiomyocytes by considering anatomy and physiology. CDK = cyclin-dependent kinase, CMs = stem cell-derived cardiomyocytes, ICD = intercalated disk, SR = sarcoplasmic reticulum, Vm = membrane potential.

Disparities exists between CMs and adult cardiomyocytes that restrict the application of CMs to disease modeling and regenerative medicine. In accordance with Alvarez-Dominguez’s definition of maturity,^[11]^ we elucidate the differences between CMs and adult cardiomyocytes by considering anatomy (e.g., form, gene circuitry, and interconnectivity) and physiology (e.g., function and proliferation) (Fig. 1).

### 2.1. Form

As CMs mature, their morphology transitions from a small, round shape to an elongated form, and their cellular volume progressively enlarges.^[14,15]^ Such morphology is crucial for cardiomyocyte contractility. Their elongated structure facilitates the assembly of longitudinally arranged myofibrils, thereby augmenting the generation of contractile forces. Concurrently, as cardiomyocytes mature, surface expansion increases capacitances, which in turn accelerates upstroke and propagation velocities of action potentials.^[2,16]^

Microscopically, the sarcomere architecture evolves throughout maturation of CMs. In nascent CMs, sarcomeres are smallish and disordered. As CMs mature, sarcomeres incrementally migrate towards T-tubules, ultimately aligning themselves perpendicularly between 2 T-tubules. Facilitated by the cytoskeletal, the sarcoplasmic reticulum and mitochondria undergo relocation, enveloping sarcomeres.^[17]^

### 2.2. Gene circuitry and function

Gene expression differences have been found in CMs and adult cardiomyocytes.^[18]^ Changes in gene expression during maturation of CMs mainly reflect the adaptability of functions.^[13,19]^ For example, the energy metabolism of CMs shifts from glycolysis to fatty acid oxidation. This is related to activation of the PGC-1/PPAR signaling pathway.^[20,21]^ The contractile function of CMs also increases with the expression and orderly arrangement of myofibrillar proteins such as myosin and actin.^[22]^ This is related to the regulation of transcription factors such as YAP1 and SF3B2.^[20]^ The electrophysiological characteristics of CMs also change with the expression and distribution of ion channels, such as potassium, sodium, and calcium channels. This is related to the role of cardiac-specific transcription factors such as NKX2.5 and GATA4.^[23]^

### 2.3. Interconnectivity

The structural basis for electromechanical coupling of the heart is the intercalated disc that connects 2 adjacent cardiomyocytes. It is critical to maintain mechanical strength of the heart and conduct electrical signals between cardiomyocytes.^[24]^ As cardiomyocytes mature, intercalated discs gradually polarize to both ends of the cell. However, the exact mechanism coordinating this process is unclear.^[25]^

### 2.4. Proliferation

Nascent CMs have a high proliferative capacity, whereas mature CMs have a poor proliferative capacity.^[26,27]^ Under regulation of the Hippo/Yap axis, mature CMs gradually exit the cell cycle, eventually stagnating in G1 phase and hardly continuing to divide.^[28,29]^ Adult cardiomyocytes have similar characteristics. After birth, DNA in cardiomyocytes replicates once, but cell division does not occur. Because of DNA ploidy, a cardiomyocyte grows in size accordingly. This is a sign of cardiomyocyte maturation. The proliferative ability of cells and the degree of maturity are usually inversely related. Thus, the maturity of cells is reflected by their proliferative ability.^[30]^

## 3. How glucose shapes cardiomyocytes

### 3.1. Glucose modulates cardiomyocyte differentiation and maturation

Glycometabolism provides energy and biosynthetic materials for cell activities, supporting the proliferation and self-renewal of stem cells. It also plays an important role in the differentiation and maturation of cardiomyocytes.

When stem cells proliferate, older mitochondria are asymmetrically distributed to 1 daughter cell, and younger mitochondria are distributed to the other.^[31]^ One cell continues to maintain stem cell characteristics, while the other acquires differentiated cell characteristics.^[32]^ In recent years, research has found that stem cells can distinguish between young and aging mitochondria during asymmetric division, and differentially allocate mitochondria of different degrees of aging to daughter cells. Young mitochondria are enriched in daughter stem cells, while aging mitochondria are enriched in differentiated daughter cells. It can be seen that the asymmetric inheritance of aging mitochondria during stem cell division is the key to maintaining the stemness of offspring stem cells. In the early stage of specific differentiation, 4 metabolic pathways, namely glycolysis, the tricarboxylic acid cycle, lipid synthesis, and glutaminolysis, are activated, providing cardiomyocytes with abundant energy and biosynthetic materials.^[33]^ Under differentiation, mitochondria gradually transform into tubular and ridge-rich structures to ensure that energy metabolism provides sufficient ATP.^[34]^ The production capacity of mitochondrion-related important enzymes and mitochondrial ROS also improve. Furthermore, glycolysis gene expression and antioxidant defense functions are inhibited.^[35–37]^

Tsogtbaatar et al^[38]^ found that changes in the metabolic pathways of pluripotent stem cells directly affect epigenetic and transcription programs, thereby affecting self-renewal. For example, glucose and glutamine metabolisms are crucial to maintain the pluripotency of human induced pluripotent stem cells (iPSCs), and metabolic enzymes and intermediates control and guide the self-renewal and differentiation of stem cells.^[39]^ By regulating glucose and glutamine metabolisms, it may optimize the culture conditions of stem cells and improve their efficiency of differentiation and maturation.

The changes in metabolic pathways during differentiation of CMs are the result of differentiation and maturation as well as active regulators. Therefore, studying the role of the glucose metabolic pathway in the differentiation and maturation of cardiomyocytes is of great significance for in-depth analysis of how glucose affects cardiomyocyte maturation.

### 3.2. Glucose affects CM maturation in vitro

GR-E14 is a cell line that responds to glucose and originates from human pluripotent stem cells. Yang et al^[40]^ found that high glucose hinders the differentiation of GR-E14 cells into cardiac cells, because it causes a significant delay in the appearance of TNNT2-positive contractile cardiomyocytes. Additionally, high glucose reduces the expression of mesodermal markers, cardiac transcription factors, mature cardiomyocyte markers, and potassium channel proteins. Consequently, high glucose compromises the function of cardiomyocytes derived from hESCs, because it reduces the frequencies of Ca^2+^ waves and contractions. Furthermore, high glucose inhibits oxidative phosphorylation and fatty acid oxidation of cardiomyocytes in human cardiac organoids, leading to an increase in nucleotide synthesis, which maintains the proliferative state of cardiomyocytes.^[41]^ Similarly, Nakano et al^[42]^ reported that high glucose inhibits the maturation of human embryonic stem cell-derived cardiomyocytes (hESC-CMs) by stimulating nucleotide biosynthesis through the pentose phosphate pathway, and proposed a novel strategy to induce cardiomyocyte maturation by lowering the glucose concentration. Immature hESC-CMs show a reduced capacity for oxidative phosphorylation and fatty acid oxidation, as well as an impaired cardiac structure and functions. Hu et al^[43]^ demonstrated that inhibition of HIF-1α and LDHA enhances the oxidative phosphorylation and fatty acid oxidation of human induced pluripotent stem cell-derived cardiomyocytes (iPSC-CMs), as well as the expression of cardiac-specific genes. This finding confirmed the positive role of oxidative phosphorylation and fatty acid oxidation in cardiomyocyte maturation, supporting the conclusions of Mills and Nakano.

In addition to directly changing the glucose concentration, altering the glucose supply mode also affects CM maturation. Yang et al^[44]^ simulated the *in vivo* starvation events that occur during prenatal-to-postnatal transition by exposing iPSC-CMs to Earle’s balanced salt solution (EBSS), EBSS formula does not contain glucose and amino acids, which enhanced their maturation *in vitro*. They found that EBSS-induced starvation activated autophagy and mitophagy in cardiomyocytes, thereby promoting CM maturation. Correia et al^[45]^ switched iPSC-CMs from glucose-supplied to fatty acid + galactose-supplied (GFAM) or fatty acid + galactose + glucose-supplied (LACM&GFAM) culture. These 2 culture modes (GFAM and LACM&GFAM) accelerated CM maturation with a higher oxidative metabolism, transcriptional signatures similar to those of adult ventricular tissue, higher myofibril density and alignment, better calcium handling, stronger contractility, and more physiological action potential kinetics.

**Figure 2.**
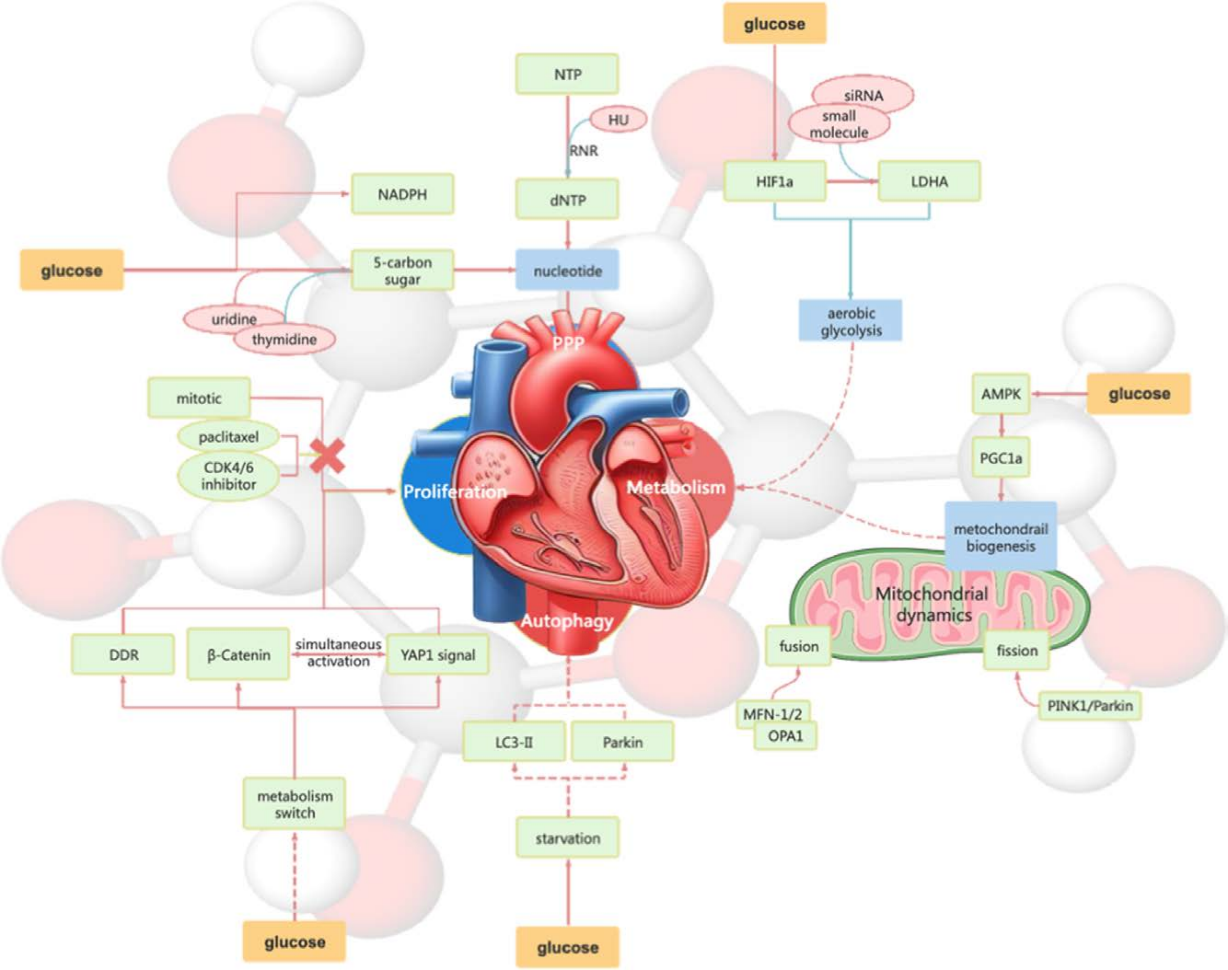
The mechanisms of the interactions between CM metabolism and maturation. AMPK = AMP-activated protein kinase, CM = stem cell-derived cardiomyocyte, DDR = DNA damage response, dNTP = deoxynucleoside triphosphates, HIF1a = hypoxia-inducible factor 1-a, HU = hydroxyurea, LC3-II = protein light chain 3-II, LDHA = lactate dehydrogenase A, MFN-1/2 = mitofusin 1/2, NADPH = nicotinamide adenine dinucleotide phosphate, NTP = nucleoside triphosphates, OPA1 = optic atrophy 1, PGC1a = peroxisome proliferator-activated receptor gamma coactivator 1-a, PPP = pentose phosphate pathway, RNR = ribonucleotide reductase, YAP1 = yes-associated protein 1.

Next, we explore the mechanisms of the interactions between CM metabolism and maturation (Fig. 2).

#### 3.2.1. Metabolic mode

When the glucose concentration decreases from 5.5 to 1 mm, expression of genes and proteins related to glycolysis also decrease, while expression of genes and proteins related to fatty acid oxidation increase in human pluripotent stem cell-derived cardiac organoids. This reflects a metabolic switch in human pluripotent stem cell-derived cardiac organoids under low glucose conditions from glycolysis-dominated to fatty acid oxidation-dominated metabolism, which is an important feature of cardiac maturation after birth.^[41]^ When the glucose concentration increases, accumulation of HIF-1α increases, which then increases expression of Glut-1 and GK, and enhances the activity of GK under hypoxia, promoting glycolysis and affecting cellular energy production.^[46]^ Upregulation of HIF-1α also activates downstream LDHA, which activates glycolysis and inhibits oxidative phosphorylation.^[43,47]^ This suggests that HIF-1α signaling is involved in the metabolic mode transition of CMs, and that this process affects CM maturation.

#### 3.2.2. Mitochondrial functions

Wang et al^[48]^ found that the mitochondrial membrane potential of iPSC-CMs exposed to high glucose (25 mM) decreases significantly, and this phenomenon is related to damage of the sarco(endo)plasmic reticulum calcium transport ATPase (SERCA) pump. Moreover, the degree of damage is positively correlated to the glucose concentration, but this process is independent of the osmotic pressure change in the culture medium. This indicates that a high glucose environment causes mitochondrial dysfunction by affecting electron transport and oxidative phosphorylation processes, which hinders the maturation process of stem cells. Garbern and Lee^[49,50]^ found that mitochondria not only produce energy, but also regulate cardiomyocyte maturation by studying the metabolic transition and mitochondrial changes during cardiomyocyte development. Mitochondria affect the phenotype of CMs by responding to and regulating the metabolic signals of CMs, thereby achieving CM maturation.

#### 3.2.3. Nucleotide biosynthesis

High glucose excessively activates the pentose phosphate pathway in CMs, which inhibits their maturation.^[51–53]^ Nakano et al^[42]^ inhibited DNA synthesis in hESC-CMs using thymidine, and found that the levels of TNNT2 and cardiac-specific transcription factor NKX2.5 were significantly increased in CMs. Addition of uridine during the culture process restored the proliferative activity of hESC-CMs. This implies that nucleotide biosynthesis is a major regulatory pathway for glucose to affect CM maturation.

#### 3.2.4. Autophagy

A large amount of autophagosome formation has been observed in the neonatal heart.^[54]^ Yang et al^[44]^ induced autophagy in hESC-CMs using EBSS to simulate in vivo starvation events by an intermittent starvation process. Through this starvation process, hESC-CMs showed a more mature structure, metabolism, and function. Structurally, the CMs had a larger cell size, more regular contractile cytoskeleton, and higher proportion of multinucleated stem cell-derived cardiomyocytes. Metabolically, EBSS treatment promoted oxidative phosphorylation in hESC-CMs. Functionally, EBSS treatment enhanced electrophysiological maturation and the calcium handling ability of hESC-CMs. This indicates that autophagy might promote CM maturation.

## 4. Normal energy metabolism of the heart and fatty acid load

As a high energy consuming organ, the heart’s energy metabolism is highly dependent on fatty acid beta oxidation (accounting for approximately 60%−90% of energy supply). The fatty acid loading status profoundly affects the energy metabolism efficiency and pathological remodeling of the heart by regulating the PGC-1 ɑ/PPAR ɑ signaling pathway. The metabolic regulation under normal physiological conditions manifests in 2 aspects: firstly, the synergistic effect of PPAR ɑ/PGC-1 ɑ. After activation, PPAR ɑ forms a complex with PGC-1 ɑ, jointly upregulating key genes such as CPT1 ɑ and CD36, promoting fatty acid uptake and mitochondrial beta oxidation, and maintaining ATP homeostasis. For example, myocardial cells convert fatty acids into ATP through this pathway, supporting the sustained contraction function of the heart. Secondly, the dynamic balance of energy metabolism. The phosphocreatine system serves as an ATP buffer pool, rapidly regulating the ATP/ADP ratio through creatine kinase to cope with fluctuations in energy demand.

The pathological effects of fatty acid overload manifest in 3 aspects: firstly, metabolic reprogramming imbalance. Long term high fatty acid environment leads to excessive activation of PPAR ɑ, inhibition of glucose utilization, resulting in decreased activity of mitochondrial respiratory chain complexes and reduced ATP production. Experimental evidence: Heart failure models show a 40% to 50% decrease in ATP content and a 2- to 3-fold increase in AMP/ATP ratio. Secondly, mitochondrial dysfunction. Fatty acid overload triggers mitochondrial fission/fusion imbalance, leading to oxidative stress and cardiomyocyte apoptosis. Thirdly, epigenetic changes. Metabolic reprogramming affects gene expression through histone modifications, inhibits cardiomyocyte proliferation, and exacerbates cardiac remodeling.^[55]^

## 5. Issues and expectations

CMs are the closest to the *in vivo* physiological state of cardiac cells. They overcome interspecies differences and can be combined with gene editing or 3D printing technologies to provide ideal tools for the exploration of disease mechanisms, toxicology evaluation, and other physiological studies.^[5,56]^ CMs can reduce unnecessary animal experiments and can be used to evaluate the functional and structural effects of drugs on the heart in a high-throughput manner, providing a good platform for efficient drug screening and preclinical evaluation. Currently, the application of CMs in the medical field mainly focuses on the following aspects.^[56–58]^ Studies of cardiac disease models: CMs are used to simulate the occurrence and development of cardiac diseases in vitro, helping researchers to understand the mechanisms of cardiac diseases more deeply. Drug screening and toxicity testing: CMs provided a more human-like physiological experimental platform. Cardiac regeneration therapy: CMs are implanted into damaged cardiac tissue, helping to repair the damaged myocardium and restore cardiac functions.

Although CMs have achieved some successes in myocardial repair, disease modeling, and drug screening, some limitations remain. We have summarized the main obstacles and potential solutions for clinical translation of CMs.^[13,22,30,58,59]^

Quality control of CMs faces some challenges. To ensure function and safety after transplantation, CMs need to have high purity, consistency, and stability. The quality of CMs can be controlled by increasing the identification of cell types, standardizing cell batches, and ensuring genetic stability.

CMs usually exhibit embryonic or neonatal characteristics, which have significant differences from adult cardiac cells in terms of morphology, structure, metabolism, and electrophysiological properties. Therefore, improving the maturity of CMs is an important research direction.

CMs face a harsh survival environment after transplantation, such as ischemia, hypoxia, inflammation, rejection, and mechanical stress. These factors cause a large amount of cell death, thereby reducing the therapeutic effect and safety. To improve the transplantation survival rate, some strategies have been proposed, such as pretreating cells, using biomaterials or carriers, enhancing angiogenesis, and immunomodulation.

CMs may cause arrhythmia after transplantation, which is a serious complication. To prevent or treat arrhythmia, some measures have been adopted, such as optimizing the selection of cell types, improving the synchrony of cells, and using electrocardiography or a pacemaker.

The clinical application prospects of CMs depend on the resolution of these obstacles. To facilitate clinical translation of CMs, we need to explore the factors and mechanisms that affect CM maturation, develop more effective quality control and pretreatment methods, and establish models and evaluation systems that are closer to the in vivo environment. The success of CM clinical translation will provide new methods to prevent and treat cardiac diseases, and improve the health and quality of life of patients.

## 6. Conclusion

In this review, we have discussed how glucose affects the maturation of CMs, which is important for cardiac development. Low glucose promotes CM maturation, but the specific molecular mechanisms of how the glucose concentration regulates the differentiation and maturation of cardiomyocytes through metabolic pathways remain unclear. Therefore, the next goal is to explore the role of glucose in cell signal transduction, especially the regulation of cardiomyocyte-specific gene expression. However, most current studies have been based on *in vitro* conditions, which might not reflect the *in vivo* environment, such as the concentration, composition, and dynamic changes of metabolic substrates, as well as the interactions with other cell types and tissues. Therefore, the results need to be validated and optimized using *in vivo* models. Clarification of the glucose effect on CM maturation may provide novel methods to enhance the quality of CMs, which will facilitate their clinical application.

Through these scientific achievements, we hope to effectively improve the maturity of CMs in a reasonable timeframe. Combined with gene editing or 3D printing technology, CMs may become the ideal tool for studying disease mechanisms, conducting drug screening and evaluation, and myocardial regeneration treatment, providing more clinical treatment choices.

## Acknowledgments

We thank Mitchell Arico from Liwen Bianji (Edanz) (https://www.liwenbianji.cn) for editing the language of a draft of this manuscript.

## Author contributions

**Conceptualization:** Junsheng Mu.

**Data curation:** Junsheng Mu.

**Formal analysis:** Junsheng Mu.

**Funding acquisition:** Junsheng Mu.

**Investigation:** Junsheng Mu.

**Project administration:** Junsheng Mu.

**Resources:** Junsheng Mu.

**Supervision:** Liqun Chi, Junsheng Mu.

**Validation:** Liqun Chi, Junsheng Mu.

**Visualization:** Liqun Chi, Junsheng Mu.

**Writing – original draft:** Liqun Chi, Junsheng Mu.

**Writing – review & editing:** Liqun Chi, Junsheng Mu.
